# The composition of the founding population of Iceland: A new perspective from 3D analyses of basicranial shape

**DOI:** 10.1371/journal.pone.0246059

**Published:** 2021-02-08

**Authors:** Kimberly A. Plomp, Hildur Gestsdóttir, Keith Dobney, Neil Price, Mark Collard

**Affiliations:** 1 Department of Archaeology, Simon Fraser University, Burnaby, British Columbia, Canada; 2 Department of Archaeology, Classics, and Egyptology, University of Liverpool, Liverpool, United Kingdom; 3 Institute of Archaeology, Reykjavik, Iceland; 4 School of Historical and Philosophical Inquiry, University of Sydney, Sydney, NSW, Australia; 5 Department of Archaeology, University of Aberdeen, Aberdeen, United Kingdom; 6 Archaeology and Ancient History, Uppsala University, Uppsala, Sweden; University at Buffalo - The State University of New York, UNITED STATES

## Abstract

The settlement of Iceland in the Viking Age has been the focus of much research, but the composition of the founding population remains the subject of debate. Some lines of evidence suggest that almost all the founding population were Scandinavian, while others indicate a mix of Scandinavians and people of Scottish and Irish ancestry. To explore this issue further, we used three-dimensional techniques to compare the basicrania of skeletons from archaeological sites in Iceland, Scandinavia, and the British Isles. Our analyses yielded two main results. One was that the founding population likely consisted of roughly equal numbers of Scandinavians and people from the British Isles. The other was that the immigrants who originated from the British Isles included individuals of southern British ancestry as well as individuals of Scottish and Irish ancestry. The first of these findings is consistent with the results of recent analyses of modern and ancient DNA, while the second is novel. Our study, therefore, strengthens the idea that the founding population was a mix of Scandinavians and people from the British Isles, but also raises a new possibility regarding the regions from which the settlers originated.

## Introduction

The permanent settlement of Iceland was one of the key events of the Viking Age, the period of northern European history between the mid 8^th^ and mid 11^th^ centuries CE that featured widespread raiding, trading, and colonisation by Scandinavians [1–3]. Though a limited degree of settlement may have occurred earlier, there is broad agreement that Iceland was not permanently colonised until the late 9^th^ century CE [4–6], and that the settlement process was rapid, with more or less all habitable areas having been occupied in 20–60 years [7–10]. However, there is still considerable uncertainty about a number of other issues. Perhaps the most important of these is the composition of the founding population.

The traditional view, based largely on written sources, is that the settlers were rich farmers and their families and followers from Norway and/or Norse settlements in the British Isles. This hypothesis has been challenged in recent decades and it is now thought that the settlement process was more complex than the written sources suggest [6, 8, 9]. The nature of the migration seems to have changed during the course of the settlement period, and there also appears to have been regional variation in the process [10–12]. Additionally, it has been argued that the size of the founding population was substantial. Vésteinsson and McGovern [10] estimated that it comprised ≥24,000 people, while Vésteinsson and Gestsdóttir [10:139] have suggested that it was probably “considerably larger” than 24,000. It has also been proposed that many of the immigrants were thralls (i.e. enslaved people) brought in to work the land, and that Icelandic society may have been characterized to some extent by a regime of serfdom rather than a series of independent farmers and their families [8, 9, 13]. Several scholars have suggested that the thralls were predominantly from Scotland and Ireland, which would mean that people who were not of Norwegian heritage may have played a significant role in the settlement of the island [4, 11, 13–21].

To untangle the complicated picture of early Icelandic settlement, including identifying who the settlers were, scholars have analysed a range of different lines of evidence, including historical documents, skeletal evidence, modern DNA, and ancient DNA (aDNA) [16, 17, 22–26]. Despite these attempts, the question of who settled Iceland has yet to be answered satisfactorily [25–29].

The documentary evidence derives mainly from two texts: *Landnámabók* (*The Book of Settlements*) and *Íslendingabók* (*The Book of Icelanders*), both of which were originally compiled in the 12^th^ century CE. A number of medieval Icelandic family sagas have also been used to shed light on the colonisation of the island [4, 6]. Together, these literary sources suggest that the founding population was primarily of Norwegian origin, and that only a very small number of non-Norwegians were involved [4]. The latter individuals are said to have been from Scotland and Ireland [4].

Morphological analyses of archaeological skeletons have yielded findings that are consistent with the literary sources [23, 30–32]. Steffensen [30, 33] and Berry [23] focused on non-metric cranial traits and concluded that the founding population of Iceland comprised individuals of Norwegian ancestry, with little or no contribution from other regions. Recently, Hallgrimsson et al. [31] revisited the issue with a similar non-metric trait dataset as Berry [23] and obtained the same result as her.

Attempts to elucidate the composition of the founding population by analysing DNA from living people have yielded results that contradict the literary evidence. Analyses of the mitochondrial and Y-chromosome DNA of modern people from Iceland, Scandinavia, and the British Isles have estimated that approximately 75% of the patrilineal ancestry of living Icelanders derives from Scandinavia, while approximately 60% of their matrilineal ancestry is from the British Isles [16], particularly Scotland and Ireland [16, 17, 34]. Interestingly, these results are similar to those obtained by Als et al. [34] in their mitochondrial DNA-based investigation of the population history of the Faroe Islands, which were settled shortly before Iceland. Als et al’s. [34] results indicated that the founding population of the Faroes also mostly comprised of men from Scandinavia and women from Scotland and Ireland.

In the last decade, several studies have analysed aDNA from Viking Age skeletons in Scandinavia and Iceland. The results of these studies have been mixed with respect to the question of the composition of Iceland’s founding population. Krzewińska et al. [21] analysed mitochondrial DNA sequences from 69 male and female skeletons from Iron Age sites in Norway and found that they showed affinities with modern Icelandic populations, a result that is consistent with the documentary evidence. Other aDNA studies have yielded results that are in line with the findings from modern DNA. For example, Ebeneserdóttir et al. [35] included Viking Age specimens from Iceland in their sample and found that around 72% of the males in their sample were most closely related to individuals from Scandinavia, while around 65% of the females were most closely related to individuals from Scotland and Ireland. Most recently, Margaryan et al. [36] analysed genomic data from the remains of 442 individuals from archaeological sites that range in date from ca. 2400 BCE to ca. 1600 CE. Among the individuals sampled were 17 from early sites in Iceland. Margaryan et al.’s [36] results suggested approximately 42% of these individuals showed UK ancestry, while *circa* 38% had Norwegian ancestry.

Unhelpfully, it is not clear which of these various hypotheses should be preferred because all the lines of evidence examined to date have shortcomings. The relevant written sources were composed centuries after the settlement period, meaning their interpretation of the events is open to question [4, 15, 25, 29, 32]. A major problem with the osteological analyses is that the use of non-metric traits has been repeatedly criticized on the grounds that scoring such traits is subjective [e.g. 37–39]. The analyses of both modern and ancient DNA relies on the genetic profiles of recent individuals, meaning that results could be affected by the drift, selection, and admixture that can be expected to have taken place over the 1000-plus years since Iceland was colonised [4]. The aDNA studies suffer from the further limitation of small sample size.

Here, we report a study designed to bring a new type of evidence to bear on the debate—three-dimensional (3D) shape data recorded on the basicrania of archaeological skeletons from Iceland, Scandinavia, and the British Isles. The suite of methods we employed is called ‘geometric morphometrics’ and has been used extensively by palaeoanthropologists to tackle comparable problems [40–44]. We focused on the shape of the basicranium because several previous studies have found that the 3D shape of this part of the cranium can be informative about relatedness among human populations [45–48]. We included specimens from sites in England as well as Scotland and Ireland in our sample on the grounds that the Vikings settled parts of southern Britain and therefore it is possible that this part of the British Isles could also have been the homeland of some members of the founding population of Iceland.

## Materials and methods

We recorded data on a total of 294 specimens from archaeological sites in Iceland, Norway, Denmark, Scotland, the Republic of Ireland, and England. Specimens were considered to be female or male based on collection records; we did not attempt to assign a sex to them anew. One-hundred-and-fifteen of the specimens were female and 181 were male. Only adult crania were included to avoid the confounding effects of ontogeny; specimens were judged to be adult based on dental eruption and epiphyseal fusion. Details of the specimens, including the sites from which they were recovered and where they are currently curated, can be found in S1 Table in S1 File.

**Fig 1:**
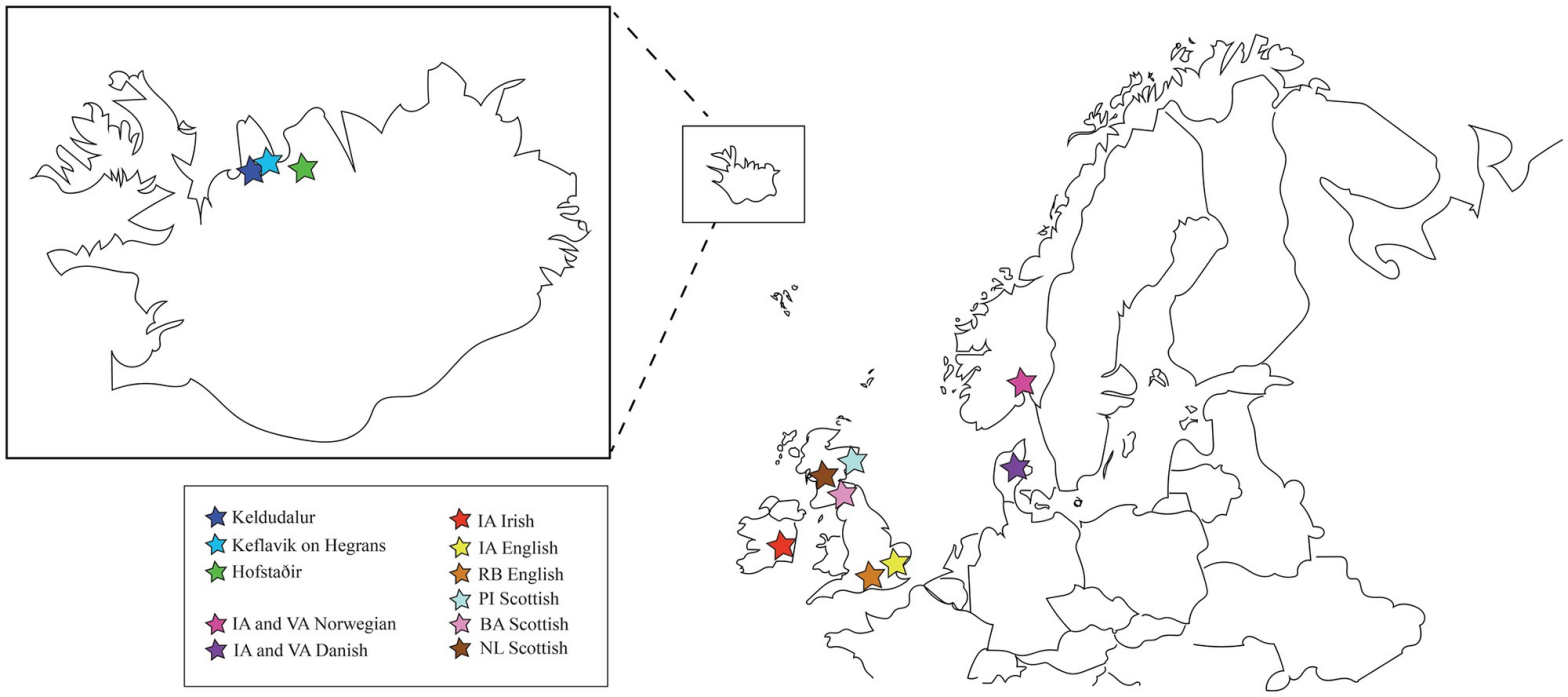
Geographic locations of the specimens included in this study. The Scandinavian specimens come from various sites throughout Norway and Denmark, thus, the general area is indicated by pink and purple stars.

The Icelandic specimens included individuals from three burial sites in northern Iceland: Keldudalur, Keflavík on Hegranes, and Hofstaðir (Fig 1). The first of these is an early 11^th^ to early 12^th^ century CE household cemetery in the Skagafjörður valley that contained 62 interments [49, 50]. We included a total of 23 specimens from this site, ten females and 13 males. Keflavík on Hegranes is also in the Skagafjörður valley and also dates from the early 11^th^ to early 12^th^ century CE. It contained 30 burials [51]. We collected data from 14 of these specimens, nine females and five males. The third cemetery is associated with the remains of a feasting hall at Hofstaðir. The cemetery and hall are located on the upper Laxá River near Lake Mývatn, and date from the late 10^th^ to early 13^th^ century CE. A total of 170 skeletons were recovered at the site [51–54]. We recorded data on 29 of them, 12 females and 17 males. Because Iceland is thought to have experienced little immigration between the end of the 10^th^ century CE and the 20^th^ century CE [26], it is likely that the majority of the individuals recovered from the three cemeteries are descendants of the islands’ settlers rather than immigrants themselves. We were unable to include specimens from sites dating to the settlement period due to access issues.

Data were collected from 29 specimens from sites in the south of Norway, including Hedmark, Sør-Trøndelag, and Telemark. We limited our sample to southern Norway in an effort to avoid inadvertently including the skeletons of Sámi. The sites date to the Iron Age and Viking Age. Eleven of the specimens are female and 17 are male.

The Danish specimens come from various sites around the country. The sites date to the either the Iron Age or the Viking Age. We measured a total of 64 specimens. These included 16 females and 48 males.

We recorded data on 20 specimens from sites in Scotland, 17 males and three females. We measured 12 males from the Pictish cemetery at Portmahomack, Scotland. The other five males and the three females originate from sites of Neolithic and Bronze Age date.

We measured 17 specimens from burials in different parts of the Republic of Ireland. The burials range in age from the Neolithic to the Iron Age. Nine of the specimens are female; eight are male.

We collected data on specimens from three sites in England: Poundbury and Maiden Castle in Dorset, and Hallet’s Garage in Kent. Poundbury and Hallet’s Garage are both Romano-British sites (3^rd^ to 4^th^ centuries CE), while Maiden Castle is an Iron Age hill fort (600 BCE to 43 CE). We measured 30 females and 34 males from Poundbury, 13 females and 18 males from Maiden Castle, and two females and four males from Hallet’s Garage. We did not include specimens from sites in southern Britain that post-date the settlement of the island by Germanic-speakers in the 5^th^ century CE to avoid the potentially confounding effects of the admixture that occurred between locals and new settlers [55].

Photogrammetry was used to generate 3D models of the crania. First, each cranium was photographed 150 times at three different angles (i.e. level with cranium, at a 15° angle, and a 40° angle) with an eight-megapixel digital SLR camera mounted with a 50mm lens. The cranium was placed on a PalaeoPi rotating table and photographs were shot at intervals of approximately 10°, following Evin et al. [56]. Next, the photographs were aligned in the 2019 release of Agisoft’s Metashape with the accuracy level set at ‘high’. Subsequently, a 3D depth map of the cranium was generated from the aligned photographs. Thereafter, a mesh model was created from the depth maps and then exported as a 3D image file.

**Fig 2:**
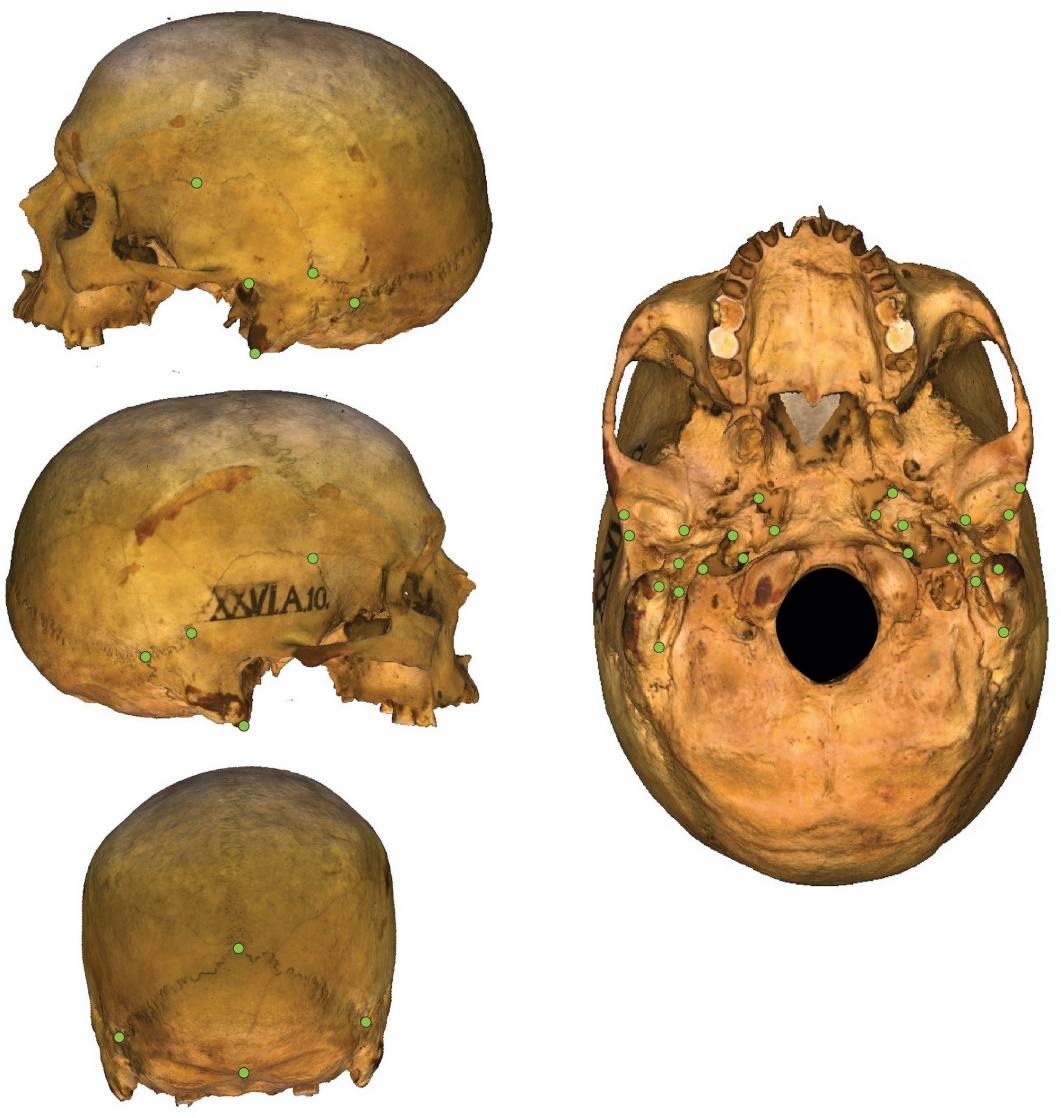
Location of the 34 landmarks used to capture the shape of the basicranium. According to Bookstein’s (1997) landmark classification scheme, there are seven Type I landmarks (lambda, asterion, auriculare, pterion, inion, carotid canal, hypoglossal canal) and 27 Type II landmarks”.

To digitize the shape of each cranial base, the 3D image file was imported into MorphoDig [57]. We recorded the X,Y,Z coordinates of a total of 34 anatomical landmarks (Fig 2). The landmarks followed those used by Harvati and Weaver [45] to capture basicranial shape. As per Bookstein’s [58] scheme, seven of the landmarks can be classified as Type I and 27 as Type II.

Intra-observer error was assessed following Neubauer et al. [59, 60]. A single cranium was digitized ten times and then Morphologika [61] was used to compare the largest Procrustes distance between the repeated landmark configurations with the smallest Procrustes distance between the non-repeated landmark configurations for all the crania. The latter distance was almost double the former. According to Neubauer et al. [59, 60], this means that intra-observer error is unlikely to have been a problem in the study.

Having assessed the intra-observer error, we divided the dataset by sex, creating a female-only dataset and separate male-only dataset. We did so to avoid the potential influence of sexual dimorphism on the results.

We then sought to minimise the impact of a number of other potential confounding factors on each sex-specific dataset. Following Klingenberg et al. [62], we reflected and re-labelled the landmark coordinates and subjected the data to generalised Procrustes analysis, which removes translational and rotational effects and scales the configurations to centroid size. We removed asymmetry by calculating the average Procrustes coordinates between the original and reflected landmarks. These procedures were carried out in MorphoJ [62]. The Procrustes data is available in the S1 File.

After addressing the confounding effects of translation, rotation, size, and asymmetry, we subjected the sex-specific datasets to three sets of analyses. In the first, we grouped the female specimens into four Operational Taxonomic Units (OTUs): *Iceland*, *Scandinavia*, *Scotland and Ireland*, and *Southern Britain* (Table 1). We assigned the specimens from England to a different OTU from the specimens from Scotland and Ireland to allow a southern British contribution to the founding population to be identified, if present. Once the OTUs were established, we subjected the Procrustes data to canonical variates analysis (CVA) to explore the similarities and differences in shape among the OTUs. Subsequently, we did the same for the male specimens. The two CVAs were performed in MorphoJ [63].

**Table 1:** Operational taxonomic units used in the study.

OTU name	Sites represented in OTU	Approximate date range of OTU	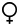	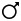
*Iceland*	All Icelandic sites represented in sample	Late 10^th^ century CE to mid 13^th^ CE	31	35
*Scandinavia*	All Norwegian and Danish sites represented in sample	Early 3^rd^ century CE to mid 11^th^ century CE	26	64
*Scotland and Ireland*	All Scottish and Irish sites represented in sample	Early 4^th^ millennium BCE to early 9^th^ century CE	12	25
*Southern Britain*	All English sites represented in sample	Early 9^th^ century BCE to early 4^th^ century CE	45	56

See the S1 File for details of the sites.

The goal of the second set of analyses was to determine whether the shape differences between the three potential source OTUs—*Scandinavia*, *Scotland and Ireland*, and *Southern Britain*—were statistically significant. To accomplish this, we subjected the Procrustes data to Principal Components Analysis (PCA) and then implemented the principal component (PC) reduction procedure outlined by Evin et al. [64, 65]. This procedure aims to reduce noise from PCs that account for little variance while still retaining all relevant shape information. It tackles this optimization problem by progressively adding PCs into a discriminant function analysis (DFA) until the cross-validation percentage begins to drop; only PCs that contribute positively to the cross-validation percentage are retained for further analysis. After carrying out Evin et al.’s [64, 65] procedure, we ran MANOVAs on the retained PC scores. As before, we did this separately with the female and male datasets. The PCAs were performed in MorphoJ [62], and both the DFAs and MANOVAs were carried out on the reduced number of PCs in R [63].

In the third set of analyses, we estimated the contribution of the three potential source OTUs to the ancestry of the Icelandic specimens. We did this by applying linear discriminant analysis (LDA) to the PCs used in the previous set of analyses. Following Evin et al. [65], we designated the potential source OTUs as the known samples and then directed the LDA to indicate which of the potential source OTUs the Icelandic specimens most likely belonged. We repeated this process 100 times for each Icelandic specimen and then calculated the average attribution percentages. A specimen was deemed to be attributed to a given source OTU if the average percentage for that OTU was ≥35%. If all the average attribution percentages for a specimen were ≤34%, it was deemed to be unattributable. Thereafter, we calculated the percentage of the Icelandic specimens that were attributed to each of the potential source OTUs. We performed one LDA for the females, and another for the males. To extrapolate from the LDA probabilities to the percentages of immigrants of Scandinavian ancestry and British Isles ancestry in Iceland’s founding population, it was necessary to make an assumption about the fitness of individuals of different ancestry. Specifically, we assumed that individuals of British Isles ancestry had the same expected number of offspring as individuals of Scandinavian ancestry. We opted for this assumption because it can be expected to result in a more conservative estimate of the number of immigrants of British Isles ancestry than the main alternative assumption, which is that, due to differences in status, individuals of Scandinavian ancestry had a higher expected number of offspring than individuals of British Isles ancestry. Under the assumption that there was no ancestry-related bias in fitness, the LDA probabilities likely reflect the composition of the founding population. We will consider the impact of this decision in the Discussion. The two LDAs were conducted in R [63].

## Results

**Fig 3:**
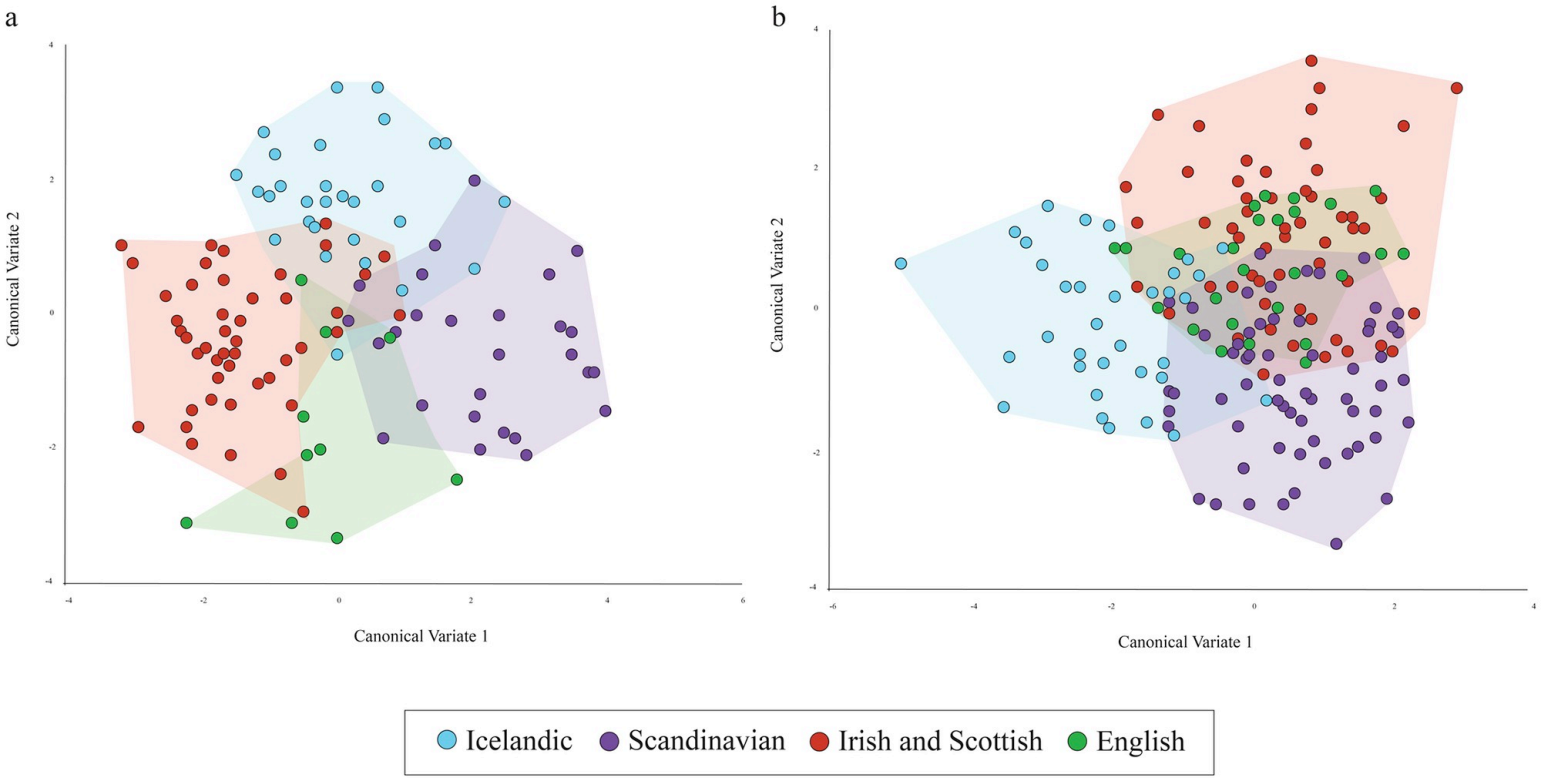
CVA scatterplots depicting the shape variation of the OTUs for (a) females and (b) males.

The female CVA yielded two CVs, which accounted for 62% and 38% of the variation, respectively. The CVs are plotted in Fig 3a. On CV1, the specimens from Iceland overlap substantially with the specimens from southern Britain, but only slightly with the specimens from Scandinavia and those from Scotland and Ireland. The situation is reversed on CV2. The Icelandic specimens overlap substantially with the specimens from Scandinavia and those from Scotland and Ireland, but only slightly with those from southern Britain.

Two CVs were also yielded by the male CVA. One accounted for 56% of the variation; the other for 44%. Fig 3b plots the two CVs. On CV1, most of the specimens from Iceland are differentiated from the specimens from the three potential source regions. On CV2, the Icelandic specimens overlap with specimens from all the potential source regions, but mostly with the specimens from southern Britain.

The PC reduction procedure retained 10 PCs for the female dataset. These PCs accounted for 68% of the total shape variance. The MANOVA for the females was significant (λ 1.3414, F = 1.5699, p = 0.0218). The pairwise MANOVAs indicated the existence of significant differences between the specimens from Scandinavia and the ones from southern Britain, but no significant differences between the specimens from Scandinavia and the ones from Scotland and Ireland, or between the specimens from southern Britain and those from Scotland and Ireland (Table 2).

**Table 2:** Results of pairwise MANOVAs.

Pairwise comparison of OTUs	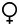	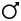
*Scandinavia* vs *Southern Britain*	λ 0.649	λ 0.513
	F = 3.351	F = 1.902
	p = 0.002	p = 0.002
*Scandinavia* vs *Scotland and Ireland*	λ 0.627	λ 0.599
	F = 1.724	F = 2.244
	p = 0.123	p = 0.07
*Southern Britain* vs *Scotland and Ireland*	λ 0.760	λ 0.724
	F = 1.455	F = 2.137
	p = 0.187	p = 0.013

Twenty PCs were retained for the male dataset. These PCs accounted for 86% of the total shape variance. The overall MANOVA for the males was significant too (λ 0.992, F = 1.9704, p<0.0001). The pairwise MANOVAs indicated that the three OTUs all differ significantly from one another (Table 2).

In the female LDA, 52% of the specimens from Iceland were assigned to *Scandinavia*, 35% to *Southern Britain*, and 10% to *Scotland and Ireland* (S2 Table in S1 File). One specimen (3%) was assigned to *Southern Britain* and *Scotland and Ireland* with equal probabilities. As such, the female LDA suggested that early Icelanders were of mixed Scandinavian-British Isles ancestry, with the Scandinavian contribution to the founding population being slightly larger than the that of the British Isles (52% *vs* 48%).

In the male LDA, the greatest number of Icelandic specimens were assigned to *Southern Britain* (34%). The remaining Icelandic specimens were evenly split between *Scandinavia* (31%) and *Scotland and Ireland* (31%) (S2 Table in S1 File). One male from Iceland was deemed to be unattributable. Thus, while the male LDA agreed with the female LDA that early Icelanders were of mixed Scandinavian-British Isles ancestry, it suggested that the British Isles contribution to the founding population was larger than the Scandinavian one (65% *vs* 31%).

## Discussion and conclusions

The study reported here focused on an important issue regarding the history of northern Europe—the composition of the founding population of Iceland. We sought to shed light on this issue by analysing 3D shape data recorded on the cranial bases of archaeological human remains from Iceland, Scandinavia, and the British Isles.

Our analyses yielded two main results. The first concerns the percentage of immigrants of Scandinavian ancestry versus the percentage of immigrants of British Isles ancestry in Iceland’s founding population. The female LDA suggested that individuals of Scandinavian ancestry slightly outnumbered individuals of British Isles ancestry, while the male LDA suggested that individuals of British Isles ancestry outnumbered individuals of Scandinavian ancestry. Thus, taken together the LDAs suggested that the founding population of Iceland consisted of roughly equal numbers of people of Scandinavian ancestry and people of British Isles ancestry.

The other noteworthy result relates to the immigrants that were of British Isles ancestry. To reiterate, we kept the specimens from sites in England separate from the specimens from sites in Scotland and Ireland to allow a southern British contribution to the founding population to be identified, if one were present. The results of the LDAs suggested that there was indeed such a contribution. Specifically, the LDAs indicated that the percentage of immigrants with southern British ancestry was 34–35%, while the percentage of those with Scottish or Irish ancestry was 10–31%. As such, the LDAs implied that there was not only a southern British contribution to the founding population, but that it was at least as large as the Scottish and Irish one.

To ensure that these findings were robust, we ran three supplementary analyses. In the first, we checked that our decision to analyse females and males separately did not bias the results. We created a mixed-sex dataset and then subjected it to LDA in the manner described previously. Thirty-eight percent of the Icelandic specimens were assigned to *Southern Britain*, 30% to *Scandinavia*, and 27% to *Scotland and Ireland*. The remaining 5% were deemed unattributable. Thus, the results of the mixed-sex analysis were similar to those obtained in the sex-specific LDAs.

In the second supplementary analysis, we ensured that our decision to combine the Norwegian and Danish specimens into a single OTU did not bias the results. We accomplished this by re-running the mixed-sex LDA with *Norway* and *Denmark* as OTUs in place of *Scandinavia*. We found that 40% of the Icelandic specimens were assigned to *Southern Britain*, 30% to *Norway*, 20% to *Scotland and Ireland*, and 10% to *Denmark*. The results of the second supplementary analysis were, therefore, also in line with the results of the main analyses.

In the third supplementary analysis, we estimated the contributions of the three OTUs to the ancestry of the founding population in a different manner. In the main analyses, we used the LDA probabilities to assign each Icelandic specimen to a single OTU and then calculated the percentage of the Icelandic specimens assigned to each of the three OTUs. This is not the only way to proceed. It is also possible to treat the LDA percentages as estimates of the contribution of the three OTUs to each Icelandic individual’s ancestry and then use the average percentages for all the Icelandic specimens as the estimates of the contributions of the three OTUs to the ancestry of the founding population. When this was done for the females, 44% of the immigrants were estimated to derive from *Scandinavia*, 33% from *southern Britain*, and 18% from *Scotland and Ireland* (S2 Table in S1 File). When it was done for the males, 37% of the founding population was estimated to derive from *Scandinavia*, 30% from *southern Britain*, and 29% from *Scotland and Ireland* (S2 Table in S1 File). Thus, estimating the contributions of the three OTUs to the ancestry of the founding population in a different way did not change either of key findings.

As we explained earlier, in order to extrapolate from the results of the LDAs to the percentages of immigrants of Scandinavian ancestry and British Isles ancestry, we assumed that there was no ancestry-related bias in fitness. To reiterate, we opted for this assumption because it resulted in a more conservative estimate of the percentage of immigrants of British Isles ancestry than the main alternative assumption, which is that, due to status differences, individuals of Scandinavian ancestry had a higher expected number of offspring than individuals of British Isles ancestry. Importantly, therefore, the LDA percentages may underestimate the number of people of Scottish, Irish, and southern British ancestry to Iceland’s founding population. If individuals of Scandinavian ancestry had a higher expected number of offspring than individuals of British Isles ancestry—due perhaps to a difference in social status—then the percentage of immigrants of Scandinavian ancestry could have been lower than the LDAs suggest.

The first of our findings—that the percentage of early Icelanders of British Isles ancestry was similar to the percentage of those of Scandinavian descent—is at odds with the picture based on the historical documents and the results of previous osteological analyses, which suggests that the people who settled Iceland were more or less exclusively from Norway [23, 30, 31, 33]. In contrast, our first finding is consistent with recent analyses of modern DNA [15–17, 34] and the most comprehensive aDNA study to have focused on the Vikings [36]. These studies also suggest that the founding population consisted of more or less equal numbers of people of Scandinavian and the British Isles ancestry [15–17, 34, 36].

There are a number of potential explanations for the discrepancy between the picture of the founding population provided by the historical documents and the one offered by the present study and recent DNA analyses. One is that the historical accounts were written long after the settlement period and therefore could have been affected by errors in the transmission of information, or bias in composition since Scandinavians most probably dominated social, political, and practical aspects of the settlement. Another potential explanation is suggested by one of Margaryan et al.’s [36] findings. These authors identified a number of cases in which an individual was buried as a Viking but was not of Scandinavian ancestry. This raises the possibility that some of settlers were people of British Isles ancestry who had assimilated into Scandinavian culture. If this were the case, Iceland’s founding population could have been a mixture of people of Scandinavian ancestry and people of British Isles ancestry but culturally Scandinavian. The discrepancy between the picture of the founding population provided by the historical documents and the one offered by the present study and the DNA studies could, therefore, be more apparent than real. It could simply be due to the authors of the historical documents having focused, perhaps unwittingly, on the settlers’ cultural identity.

The discrepancy between the results of the previous osteological analyses and the findings of the present study is more puzzling, given that all the analyses in question involved human skeletal data. One potential explanation is that the earlier osteological analyses simply did not include the remains of early Icelanders with British Isles ancestry due to the vagaries of sampling. Another possibility is that the data analysed previously—i.e. non-metric traits—are unreliable for tracing genetic relationships. As mentioned in the Introduction, use of non-metric osteological traits has been repeatedly criticised on the grounds that the scoring of such traits is subjective [e.g. 37–39]. A third possibility is that some non-metric traits are informative about relatedness among human populations but others are not, and a sufficient number of the latter were included in the previous studies to obscure the contribution of individuals from the British Isles to the founding population.

Our second finding—that there was a southern British contribution to the founding population that was at least as large as the Scottish and Irish one—does not have a precedent in the literature, to the best of our knowledge. We have yet to find a published study in which it is suggested that people from southern Britain participated in the settlement of Iceland in substantial numbers. Studies in which non-Scandinavians have been found to be among the settlers have concluded that the people in question were from Scotland and Ireland [16, 17, 22, 34], the UK [15], or the British Isles in general [35, 36]; they have not specifically identified southern Britain as a source of immigrants.

While our finding regarding the involvement of people from southern Britain in the settlement of Iceland is novel, it should not be particularly surprising. It is well-established that the Vikings raided southern Britain, including the area in which our southern British specimens were recovered, during the early part of the Viking Age [66–69]. Indeed, traditionally the 793 CE Viking attack on Holy Island in Northumberland was regarded as the beginning of the Viking Age [4]. If obtaining slaves was a major component of the raids, as seems to be the case [19, 70], then it follows that a large number of people could have been enslaved during the many raids on southern Britain, and some of these individuals could have been transported to Iceland. Interestingly, Dr. Alex Woolf of St. Andrews University believes that *Landnámabók* contains at least one passage that points to the presence of people of southern British ancestry among the settlers [personal communication, 15^th^ December 2020]. The passage occurs in Chapter 81, and discusses a woman called Vilborg, who was married to Thord Skeggi. Importantly for present purposes, Vilborg is reported to have been the daughter of Oswald and Wulfrun, the daughter of Eadmund. Dr. Woolf thinks that Vilborg’s father was probably the King Oswald who ruled East Anglia in the 1870s, and that Vilborg’s maternal grandfather was likely the King Eadmund who ruled East Anglia in the 850s and 860s and later became Saint Edmund the Martyr.

We noted earlier that analyses of mtDNA and Y-chromosome DNA have estimated that around three-quarters of the patrilineal ancestry of living Icelanders is from Scandinavia, while about two-thirds of their matrilineal ancestry originates from the British Isles [15–17, 34, 36]. Unfortunately, our analyses were unable to cast further light on this issue. While there were marked differences between the results of female and male LDAs, it is unlikely that they reflect differences in the relative numbers of females and males from the potential source populations. Because our Icelandic specimens are almost certainly descendants of settlers rather than original settlers themselves, the only way the aforementioned LDA results could be informative about sex differences in ancestry is if the inheritance of basicranial shape is sex linked and there is no reason to think that is the case. Thus, the differences between our female and male LDA results are likely the result of random variation in preservation and sampling and, therefore, are not meaningful.

With regard to future research, there are two obvious next steps. One is to repeat the study with a more comprehensive sample. Our Icelandic sample was limited to skeletons from three burial sites, all of which postdate the conversion to Christianity. More than 50 sites in Iceland have yielded human skeletal remains that predate the conversion, and it would be desirable to include as many of these specimens as possible in a follow-up study. It would also be productive to include specimens from other regions of Norway. Our study included only a few individuals from Southwest Norway, which would be a particularly good region to target for more specimens because *Landnámabók* suggests that many settlers departed from there [71]. Lastly, it would be useful to include specimens from other regions of the British Isles. An important gap in our sample is the Northern Isles of Scotland, i.e. the archipelagos of Orkney and Shetland. The Northern Isles were under Norse control for several centuries and are geographically close to Iceland. As such, they are an obvious potential additional homeland for Iceland’s settlers.

The other obvious next step is to repeat the study with data from one or more other regions of the skull. To reiterate, we focused on the basicranium because previous analyses involving human crania have shown that 3D basicranial shape correlates significantly with among-population genetic distance. Accordingly, we reasoned that focusing on the basicranium was likely to shed light on the ancestry of the Icelandic specimens in our sample. However, work in palaeoanthropology has suggested that there are not major differences among the cranial regions with regard to inferring ancestry [e.g. 72]. With this in mind, it would be useful to apply 3D geometric morphometric techniques to the faces, cranial vaults, and/or mandibles of a large sample of suitably dated Icelandic, Scandinavian, and British Isles specimens.

## Supporting information

S1 File (DOCX)
