# Correction: The composition of the founding population of Iceland: A new perspective from 3D analyses of basicranial shape

**DOI:** 10.1371/journal.pone.0249393

**Published:** 2021-03-25

**Authors:** The PLOS ONE staff

S2 File is omitted from the list of Supporting Information; the publisher apologizes for the error. It can be viewed below.

## Supporting information

S2 File Supplementary dataset. This file includes supplementary data.
(XLSX)
