# Supplementary material for: The composition of the founding population of Iceland: A new perspective from 3D analyses of basicranial shape
Source: PLoS One. 2021 Feb 8;16(2):e0246059. doi: 10.1371/journal.pone.0246059 (PMC7870008; doi:10.1371/journal.pone.0246059)
Supplement: S1 File — (DOCX) [file pone.0246059.s001.docx]

**Supplementary Information for Plomp, Gestsdóttir, Dobney, Price, and Collard’s ‘The composition of the founding population of Iceland:**

**A new perspective from 3D analyses of basicranial shape’**

**Table S1. Details of individuals included in the analyses.**

| **Country** | **Site** | **Collection number (individual)** |
| --- | --- | --- |
| Iceland | Hofstaðir | 21 |
|  | Hofstaðir | 11 |
|  | Hofstaðir | 19 |
|  | Hofstaðir | 39 |
|  | Hofstaðir | 54 |
|  | Hofstaðir | A9 |
|  | Hofstaðir | 1 |
|  | Hofstaðir | 3 |
|  | Hofstaðir | 4 |
|  | Hofstaðir | 8 |
|  | Hofstaðir | 10 |
|  | Hofstaðir | 13 |
|  | Hofstaðir | 14 |
|  | Hofstaðir | 17 |
|  | Hofstaðir | 20 |
|  | Hofstaðir | 45 |
|  | Hofstaðir | 47 |
|  | Hofstaðir | 51 |
|  | Hofstaðir | 52 |
|  | Hofstaðir | 53 |
|  | Hofstaðir | 57 |
|  | Hofstaðir | 65 |
|  | Hofstaðir | 76 |
|  | Hofstaðir | 87 |
|  | Hofstaðir | 98 |
|  | Hofstaðir | 26 |
|  | Hofstaðir | 27 |
|  | Hofstaðir | 29 |
|  | Hofstaðir | 38 |
|  | Keldudalur | 5 |
|  | Keldudalur | 11 |
|  | Keldudalur | Com |
|  | Keldudalur | Gg |
|  | Keldudalur | G2 |
|  | Keldudalur | M1 |
|  | Keldudalur | S1 |
|  | Keldudalur | S6 |
|  | Keldudalur | S14 |
|  | Keldudalur | 4 |
|  | Keldudalur | 10 |
|  | Keldudalur | 14 |
|  | Keldudalur | 19 |
|  | Keldudalur | 20 |
|  | Keldudalur | 22 |
|  | Keldudalur | 25 |
|  | Keldudalur | 29a |
|  | Keldudalur | 30 |
|  | Keldudalur | 29b |
|  | Keldudalur | 8 |
|  | Keflavík on Hegranes | 7 |
|  | Keflavík on Hegranes | 9 |
|  | Keflavík on Hegranes | 39 |
|  | Keflavík on Hegranes | 1 |
|  | Keflavík on Hegranes | 3 |
|  | Keflavík on Hegranes | 20 |
|  | Keflavík on Hegranes | 25 |
|  | Keflavík on Hegranes | 29 |
|  | Keflavík on Hegranes | 35 |
|  | Keflavík on Hegranes | 40 |
|  | Keflavík on Hegranes | 41 |
|  | Keflavík on Hegranes | 49 |
|  | Keflavík on Hegranes | 12 |
|  | Keflavík on Hegranes | 30 |
| Norway | Enkoping | 1653 |
|  | Enkoping | 1652 |
|  | Enkoping | 1651 |
|  | Evenskjar | 1540 |
|  | Finndal Solim, Telemark | 1508 |
|  | Gjelvoll Orlandet | 3706 |
|  | Krigsgraber D Agder | 4721 |
|  | Kaupang | 5280 |
|  | Kirkebakken, Utaug Orlandet | 5278 |
|  | Ulhang Orland Sorxonelag | 5304 |
|  | Megarden Haltsal | 4564 |
|  | Nedre Eines Lindesis | 4007 |
|  | Oharminak Oleogexina | 4008 |
|  | Stonfosen gods | 4407 |
|  | Sor Trondeley | 4565 |
|  | Tjeldsun, Vikintid | 4727 |
|  | Vando | 2815 |
|  | Vando | 2814 |
|  | Vando | 2816 |
|  | Vando | 2817 |
|  | Harberg Orlandet Sor Tromelang | 3983 |
|  | Hindbohmen Tysfjord | 1513 |
|  | Klepp Jaeren | 1537 |
|  | Kamberg Humedal Stensen | 1526 |
|  | Vinge Felemarken | V1 |
|  | Lind Leinanger Olonland | 1511 |
|  | Vando | 2853 |
|  | Orlandet | 4764 |
|  | Ostrax Orland | 4481 |
|  | Skeie Klepp Rogland | 7087 |
|  | Ulhang Orland Sorxonelag | 4503 |
| Demark | Lille Vasby | 19 |
|  | Krongmerken | 12 |
|  | Krongmerken | 4006 |
|  | Simonsborg | S1 |
|  | Simonsborg | S2 |
|  | Simonsborg | S5 |
|  | Simonsborg | S9 |
|  | Simonsborg | S14 |
|  | Simonsborg | S31 |
|  | Varpelev | 5 |
|  | Varpelev | E |
|  | Varpelev | J |
|  | Varpelev | N |
|  | Varpelev | J |
|  | Vester Egesborg | Ve1 |
|  | Vester Egesborg | VeA |
|  | Hesselbjerg | 2 |
|  | Hesselbjerg | A |
|  | Hesselbjerg | Gb |
|  | Hesselbjerg | G5 |
|  | Hesselbjerg | G6 |
|  | Hesselbjerg | G30 |
|  | Hesselbjerg | Ur |
|  | Hesselbjerg | 4005 |
|  | Hesselbjerg | 4 |
|  | Hesselbjerg | Kl |
|  | Simonsborg | S25 |
|  | Simonsborg | S26 |
|  | Simonsborg | S27 |
|  | Simonsborg | Sh |
|  | Skyttemarksej | 64 |
|  | Senderumgaard | Stby |
|  | Toksvaerd By | Tby |
|  | Varpelev | V3 |
|  | Varpelev | V82 |
|  | Varpelev | 1645 |
|  | Varpelev | VV1 |
|  | Hesselbjerg | HJ |
|  | Smide | 3983 |
|  | Bakkendrub | 1513 |
|  | Trelleborg | 1537 |
|  | Bogøvej | Bff |
|  | Bogøvej | BAD |
|  | Farevjlegard | Fal |
|  | Galgedi | Galz |
|  | Galgedi | Gbgq |
|  | Galgedi | Gta |
|  | Galgedi | Guo |
|  | Galgedi | Gaxe |
|  | Galgedi | Gls |
|  | Hesselbjerg | Hesj |
|  | Hessum | Hessm |
|  | Kaagarden | Kel |
|  | Kaagarden | Kap |
|  | Kaagarden | Kay |
|  | Kaagarden | Nbh |
|  | Kaagarden | Kmlr |
|  | Kumle Hoje | Khh |
|  | Krongmarken | Kr16 |
|  | Krongmarken | Kr61 |
|  | Krongmarken | Kr67 |
|  | Krongmarken | Kr314 |
|  | Treleeborg | Tab |
|  | Treleeborg | Tsb8 |
| Southern Britain | Poundbury | 15 |
|  | Poundbury | 94 |
|  | Poundbury | 100 |
|  | Poundbury | 107 |
|  | Poundbury | 110 |
|  | Poundbury | 114 |
|  | Poundbury | 118 |
|  | Poundbury | 119 |
|  | Poundbury | 126 |
|  | Poundbury | 142 |
|  | Poundbury | 143 |
|  | Poundbury | 144 |
|  | Poundbury | 155 |
|  | Poundbury | 177 |
|  | Poundbury | 185 |
|  | Poundbury | 207 |
|  | Poundbury | 212 |
|  | Poundbury | 255 |
|  | Poundbury | 276 |
|  | Poundbury | 284 |
|  | Poundbury | 286 |
|  | Poundbury | 290 |
|  | Poundbury | 298 |
|  | Poundbury | 309 |
|  | Poundbury | 322 |
|  | Poundbury | 381 |
|  | Poundbury | 392 |
|  | Poundbury | 393 |
|  | Poundbury | 398 |
|  | Poundbury | 401 |
|  | Poundbury | 403 |
|  | Poundbury | 500 |
|  | Poundbury | 566 |
|  | Poundbury | 568 |
|  | Poundbury | 574 |
|  | Poundbury | 625 |
|  | Poundbury | 638 |
|  | Poundbury | 642 |
|  | Poundbury | 644 |
|  | Poundbury | 650 |
|  | Poundbury | 654 |
|  | Poundbury | 658 |
|  | Poundbury | 679 |
|  | Poundbury | 684 |
|  | Poundbury | 707 |
|  | Poundbury | 1004 |
|  | Poundbury | 1022 |
|  | Poundbury | 1030 |
|  | Poundbury | 1095 |
|  | Poundbury | 1225 |
|  | Poundbury | 19 |
|  | Poundbury | 31 |
|  | Poundbury | 103 |
|  | Poundbury | 109 |
|  | Poundbury | 113 |
|  | Poundbury | 121 |
|  | Poundbury | 208 |
|  | Poundbury | 221 |
|  | Poundbury | 247 |
|  | Poundbury | 277 |
|  | Poundbury | 305 |
|  | Poundbury | 385 |
|  | Poundbury | 515 |
|  | Poundbury | 352 |
|  | Hallett’s Garage | 1347 |
|  | Hallett’s Garage | 1519 |
|  | Hallett’s Garage | 1887 |
|  | Hallett’s Garage | 1983 |
|  | Hallett’s Garage | 2045 |
|  | Maiden Castle | 64 |
|  | Maiden Castle | 69 |
|  | Maiden Castle | 78 |
|  | Maiden Castle | 80 |
|  | Maiden Castle | 92 |
|  | Maiden Castle | 94 |
|  | Maiden Castle | 95 |
|  | Maiden Castle | 97 |
|  | Maiden Castle | 98 |
|  | Maiden Castle | 101 |
|  | Maiden Castle | 105 |
|  | Maiden Castle | 110 |
|  | Maiden Castle | 112 |
|  | Maiden Castle | 61 |
|  | Maiden Castle | 62 |
|  | Maiden Castle | 71 |
|  | Maiden Castle | 115 |
|  | Maiden Castle | 63 |
|  | Maiden Castle | 74 |
|  | Maiden Castle | 75 |
|  | Maiden Castle | 76 |
|  | Maiden Castle | 77 |
|  | Maiden Castle | 79 |
|  | Maiden Castle | 90 |
|  | Maiden Castle | 99 |
|  | Maiden Castle | 102 |
|  | Maiden Castle | 104 |
|  | Maiden Castle | 959 |
|  | Maiden Castle | 60 |
|  | Maiden Castle | 70 |
|  | Maiden Castle | 89 |
| Scotland | Portmahomack | 30 |
|  | Portmahomack | 31 |
|  | Portmahomack | 36 |
|  | Portmahomack | 37 |
|  | Portmahomack | 85 |
|  | Portmahomack | 90 |
|  | Portmahomack | 91 |
|  | Portmahomack | 112 |
|  | Portmahomack | 113 |
|  | Portmahomack | 128 |
|  | Portmahomack | 147 |
|  | Portmahomack | 148 |
| Scotland | Broomend of Criche | Br5 |
|  | Broomend of Criche | Br6 |
|  | Broomend of Criche | BrD2 |
|  | Broomend of Criche | Brlb |
|  | Broomend of Criche | Bromw |
|  | Dounreay | Dr252 |
|  | Cockenzie | C21 |
|  | Lesmurdie | L14 |
| Republic of Ireland | Carrow Keel | AC |
|  | Lough Gur | 2904 |
|  | No info available | 2337 |
|  | No info available | Ai |
|  | No info available | B385 |
|  | Lough Gur | CH |
|  | Lough Gur | Gt |
|  | No info available | 966 |
|  | No info available | 972 |
|  | No info available | 977 |
|  | No info available | 986 |
|  | No info available | 2257 |
|  | Lough Gur | Ph |
|  | Lough Gur | Wb |

**Table S2. LDA results for Icelandic individuals.**

|  | 1^st^ probability | OTU designation | 2^nd^ probability | OTU designation |
| --- | --- | --- | --- | --- |
| Females | | | | |
| Keldudalur |  | | | |
| KEH-30 | 70% | Scandinavia | 30% | Southern Britain |
| KEH-19 | 60% | Scandinavia | 30% | Southern Britain |
| KEH-14 | 47% | Southern Britain | 47% | Scandinavia |
| KEH-10 | 67% | Scandinavia | 20% | Scotland and Ireland |
| KEH-4 | 77% | Scandinavia | 20% | Scotland and Ireland |
| KEH-S-14 | 53% | Scandinavia | 40% | Southern Britain |
| KEH-g2 | 77% | Scotland and Ireland | 23% | Scandinavia |
| KEH-gg | 97% | Scandinavia | 3% | Southern Britain |
| KEH-A-29 | 60% | Scandinavia | 40% | Southern Britain |
| KEH-5 | 60% | Southern Britain | 33% | Scandinavia |
| Keflavik on Hegranes |  |  |  |  |
| KEF-7 | 67% | Scandinavia | 33% | Southern Britain |
| KEF-30 | 83% | Scandinavia | 30% | Southern Britain |
| KEF-12 | 47% | Southern Britain | 27% | Scotland and Ireland |
| KEF-29 | 60% | Scandinavia | 40% | Southern Britaim |
| KEF-20 | 53% | Scandinavia | 40% | Scotland and Ireland |
| KEF-3 | 50% | Southern Britain | 43% | Scandinavia |
| KEF-1 | 87% | Scandinavia | 13% | Southern Britain |
| KEF-39 | 70% | Southern Britain | 30% | Scandinavia |
| KEF-9 | 60% | Scandinavia | 40% | Southern Britain |
| Hofstaðir |  |  |  |  |
| HSM-21 | 50% | Scotland and Ireland | 50% | Southern Britain |
| HSM-27 | 43% | Southern Britain | 37% | Scotland and Ireland |
| HSM-26 | 70% | Scotland and Ireland | 30% | Southern British |
| HSM-52 | 90% | Scotland and Ireland | 7% | Scandinavian |
| HSM-14 | 73% | Scotland and Ireland | 23% | Scotland and Ireland |
| HSM-13 | 53% | Scandinavia | 23% | Scotland and Ireland |
| HSM-10 | 50% | Southern Britain | 47% | Scotland and Ireland |
| HSM-4 | 70% | Southern Britain | 20% | Scandinavia |
| HSM-3 | 97% | Scandinavia | 3% | Southern Britain |
| HSM-1 | 67% | Scandinavia | 30% | Southern Britain |
| HSM-19 | 57% | Southern Britain | 43% | Scotland and Ireland |
| HSM-11 | 63% | Scandinavia | 30% | Southern Britain |
| Males | | | | |
| Keldudalur |  |  |  |  |
| KEH-A -29 | 53% | Scotland and Ireland | 47% | Southern Britain |
| KEH-11 | 62% | Scotland and Ireland | 28% | Scandinavia |
| KEH-com | 97% | Scandinavia | 3% | Scotland and Ireland |
| KEH-M-1 | 88% | Scotland and Ireland | 12% | Scandinavia |
| KEH-S-1 | 72% | Scandinavia | 25% | Southern Britain |
| KEH-S-6 | 97% | Scandinavia | 3% | Southern Britain |
| KEH-20 | 75% | Southern Britain | 19% | Scotland and Ireland |
| KEH-22 | 97% | Scandinavia | 3% | Scotland and Ireland |
| KEH-25 | 56% | Scandinavia | 44% | Southern Britain |
| KEH-34 | 59% | Scotland and Ireland | 31% | Scandinavia |
| KEH-13 | 88% | Southern Britain | 6% | Scotland and Ireland |
| KEH-21 | 63% | Southern Britain | 34% | Scandinavia |
| KEH-8 | 94% | Scandinavia | 6% | Scotland and Ireland |
| Keflavik on Hegranes |  |  |  |  |
| KEF-25 | 56% | Southern Britain | 44% | Scandinavia |
| KEF-35 | 56% | Southern Britain | 41% | Scotland and Ireland |
| KEF-40 | 63% | Southern Britain | 31% | Scandinavia |
| KEF-41 | 50% | Southern Britain | 44% | Scandinavia |
| KEF-49 | 81% | Scotland and Ireland | 19% | Southern Britain |
| Hofstaðir |  |  |  |  |
| HSM-39 | 91% | Scandinavia | 9% | Southern Britain |
| HSM-54 | 72% | Southern Britain | 28% | Scotland and Ireland |
| HSM-A-9 | 100% | Scotland and Ireland | - | - |
| HSM-8 | 47% | Scotland and Ireland | 47% | Scandinavia |
| HSM-17 | 81% | Scotland and Ireland | 9% | Southern Britain |
| HSM-20 | 81% | Scandinavia | 19% | Southern Britain |
| HSM-45 | 88% | Scandinavia | 9% | Scotland and Ireland |
| HSM-47 | 53% | Southern Britain | 38% | Scotland and Ireland |
| HSM-51 | 38% | Scotland and Ireland | 38% | Southern Britain |
| HSM-53 | 62% | Scandinavia | 31% | Southern Britain |
| HSM-57 | 94% | Southern Britain | 6% | Scandinavia |
| HSM-65 | 41% | Scotland and Ireland | 31% | Scandinavia |
| HSM-76 | 69% | Scandinavia | 31% | Southern Britain |
| HSM-87 | 71% | Scandinavia | 28% | Scotland and Ireland |
| HSM-98 | 63% | Southern Britain | 25% | Scotland and Ireland |
| HSM-29 | 53% | Scotland and Ireland | 47% | Southern Britain |
| HSM-38 | 100% | Scotland and Ireland | - | - |
